# Functional identification of *PGM1* in the regulating development and depositing of inosine monophosphate specific for myoblasts

**DOI:** 10.3389/fvets.2023.1276582

**Published:** 2023-12-18

**Authors:** Wei Zhao, Zhengyun Cai, Chuanhao Wei, Xiaoping Ma, Baojun Yu, Xi Fu, Tong Zhang, Yaling Gu, Juan Zhang

**Affiliations:** College of Animal Science and Technology, Ningxia University, Yinchuan, China

**Keywords:** Jingyuan chicken, inosine monophosphate, *PGM1* gene, purine metabolism, meat flavor

## Abstract

**Background:**

Inosine monophosphate (IMP) is naturally present in poultry muscle and plays a key role in improving meat flavour. However, IMP deposition is regulated by numerous genes and complex molecular networks. In order to excavate key candidate genes that may regulate IMP synthesis, we performed proteome and metabolome analyses on the leg muscle, compared to the breast muscle control of 180-day-old Jingyuan chickens (hens), which had different IMP content. The key candidate genes identified by a differential analysis were verified to be associated with regulation of IMP-specific deposition.

**Results:**

The results showed that the differentially expressed (DE) proteins and metabolites jointly involve 14 metabolic pathways, among which the purine metabolic pathway closely related to IMP synthesis and metabolism is enriched with four DE proteins downregulated (with higher expression in breast muscles than in leg muscles), including adenylate kinase 1 (*AK1*), adenosine monophosphate deaminase 1 (*AMPD1*), pyruvate kinase muscle isoenzyme 2 (*PKM2*) and phosphoglucomutase 1 (*PGM1*), six DE metabolites, Hypoxanthine, Guanosine, L-Glutamine, AICAR, AMP and Adenylsuccinic acid. Analysis of *PGM1* gene showed that the high expression of *PGM1* promoted the proliferation and differentiation of myoblasts and inhibited the apoptosis of myoblasts. ELISA tests have shown that *PGM1* reduced adenosine triphosphate (ATP) and IMP and uric acid (UA), while enhancing the biosynthesis of hypoxanthine (HX). In addition, up-regulation of *PGM1* inhibited the expression of purine metabolism pathway related genes, and promoted the IMP *de novo* and salvage synthesis pathways.

**Conclusion:**

This study preliminarily explored the mechanism of action of *PGM1* in regulating the growth and development of myoblasts and specific IMP deposition in Jingyuan chickens, which provided certain theoretical basis for the development and utilization of excellent traits in Jingyuan chickens.

## Background

Poultry has become the second largest consumer meat in China, its status is second only to pork (1, 2) and the supply and demand of poultry meat shows an overall upward trend (3, 4). In recent years, with the rapid development of animal husbandry and the continuous improvement of people’s living standards, consumers not only pursue the nutritional value of poultry meat, but also have higher requirements for meat flavor. Compared with beef and pork, chicken is deeply loved by consumers because of its compact meat quality, unique flavor, excellent taste, high nutritional value and so on (5). Over the past decades, research on poultry has focused on improving its growth rate, feed conversion and meat production (6). It was found that after selective breeding, the growth rate of poultry was significantly increased, but the meat quality was decreased, which could not correspond to the consumer demand for chicken meat (7, 8). Therefore, under the premise of ensuring the growth rate of poultry, improving the meat flavor and cultivating high-quality local chicken breeds with excellent breeds and unique flavors has become an important challenge for modern molecular breeding.

Inosine monophosphate (IMP) is widely regarded as an important indicator for evaluating the flavor of livestock and poultry meat and it is the main substance affecting muscle flavor and texture, as well as an important factor determining the economic value of chicken meat. Breeders regard it as a current research hotspot (9, 10). IMP first appeared in beef and was not proven to have fresh flavor until 1913 (11). When IMP undergoes the maillard reaction, it produces various volatile aromatic compounds that enhance sweetness and suppress acidity and bitterness in food (12). Studies have concluded that genetics, environment, feeding practices and nutritional levels have a great influence on the specific deposition of IMP, with genetics being the most important factor (13, 14). Analysis of three single nucleotide polymorphisms (SNPs) in the *AMPD1* gene of fast-partridge and lingcock showed that the lingcock homozygous genotype AA had significantly higher IMP content than the GG genotype at loci 4,064 and 6,805, whereas the fast-partridge chicken genotype had significantly higher IMP content than the AA genotype at loci 6,805. SNP 6,805A/G can be used as a possible candidate marker for IMP content in fast partridge and Lingshan chicken (15). Fifteen key co-expression genes which may regulate IMP metabolism were screened from the leg muscle of Rugao chicken, a famous local breed in China. The correlation coefficients between 19 IMP genes and 15 co-expression genes were calculated and the regulatory network was constructed (16). Yu conducted transcriptome analysis of breast muscle and leg muscle of Jingyuan chicken and found 39 differential miRNAs and 666 differential mRNAs between breast muscle and leg muscle and 29 miRNA target gene pairs. Correlation analysis showed that gga-miR-107-3p-KLHDC2 negative interaction may be the key regulator of IMP deposition (17).

Phosphoglucose-converting enzyme 1 (*PGM1*) is a key enzyme in glycolysis, gluconeogenesis and catabolism and is widely found in animals, plants and microorganisms. It belongs to the α-D-glucose convertase superfamily and is a key regulator of carbohydrate metabolism in mammals. It has been shown that *PGM1* catalyzes the reversible conversion between α-D-glucose-1-phosphate and α-D-glucose-6-phosphate by bisphosphorylating the sugar intermediate α-D-glucose-1-bisphosphate. Meanwhile, *PGM1* also catalyzes the reversible interconversion of glucose 6-phosphate (G-6-P) and glucose 1-phosphate (G-1-P) (18). In addition, in the pentose phosphate pathway, G-6-P forms ribose-5-phosphate in the presence of *PGM1* and ribose-5-phosphate reacts with ATP to form ribulose 1-pyrophosphate-5-phosphate, followed by the formation of IMP, suggesting that *PGM1* plays an important role in the synthesis of IMP (19). It has been pointed out that the *PGM1* gene is located on the long arm of porcine chromosome 6, in the same chromosomal region as many key genes affecting meat flavor and pork growth and is closely related to the growth and development of animal muscle (20, 21). However, the effect of the *PGM1* gene on growth and development and meat quality traits in poultry is unclear.

The aim of this study was to reveal the mechanism of *PGM1* regulating IMP-specific deposition in Jingyuan chickens and to provide reference information for the development and utilization of excellent traits in Jingyuan chickens.

## Materials and methods

### Experiment material

The Jingyuan chickens used in this study were all from the Jingyuan Chicken National Conservation Farm (Pengyang, Ningxia). Chicks of Jingyuan chickens with the same genetic background hatched in the same batch were selected and fed with the same food in 40 × 40 × 40 cm cages. The chicks were reared to 180 d, weighing 2.5 ± 0.23㎏, fasted for 12 h before slaughter and tissue samples were collected from the breast and leg muscles of 15 Jingyuan chicken hens in three replicates after bloodletting and slaughter, which were quickly put into liquid nitrogen tanks. High performance liquid chromatography (HPLC) was used to determine the IMP content in breast and leg muscle tissues (22) (Supplementary Table S1). Breast muscle and leg muscle with difference IMP content were selected for proteome and metabolome analyses.

### Combined analysis of proteomics and metabolomics

The proteome analyses raw data are early data from the laboratory and used the breast muscle as the control group and the leg muscle as the experimental group for proteome analyses analysis (23). In short, the sample quality meets the analyses requirements. Through preliminary verification, with *p* < 0.01 as the threshold of significance and Foldchange >1.2 or Foldchange <1/1.2 as the screening condition for DE proteins, 101 DE proteins were screened out and the number of DE proteins for each individual is shown in Supplementary Table S2. Subsequently, using *p* < 0.01 and VIP (Variable Importance of Projection) > 1 as screening criteria, all DE metabolites between the breast and leg muscles of Jingyuan chicken were screened in both positive ion mode and negative ion mode modes. KEGG (https://www.kegg.jp/kegg/pathway.html) was used for enrichment analysis of DE metabolite related pathways.

### Cell transfection

The pcDNA3.1-*PGM1* overexpression vector was synthesized (Promega, Madison, WI, United States) from Zhongke Yutong (Shanxi, China), while siRNA-*PGM1* (F: CAAGAUGUUUGUUGU GUGUGUGUGA; R:UCAUACAAAGCAAUCUUUG) was designed and synthesized by Jisai (Guangzhou, China). The myoblasts were cultured in 20% FBS (Cell Max, Lanzhou, China) + 79% DMEM/F12 (BI, Israel) + 1% Double antibody (G-clone, Beijing, China) growth medium until the cell density reached about 70%, starved for 2 h and transfected with transfection reagents (Zeta Life, United States).

### Real-time fluorescence quantitative PCR

The total RNA of myoblasts was extracted by trizol method (Takara, Japan). The integrity and concentration of total RNA were detected by 1% gel electrophoresis and Nanodrop ND-2000 ultramicro spectrophotometer (Therom, United States). According to the manufacturer’s instructions, the reverse transcription kit (Novizan, United States) was used to synthesize cDNA. According to the mRNA sequence of gene and actin (β-actin) provided by NCBI database (https://www.ncbi.nlm.nih.gov/), gene primers were designed by Primer Premier 5.0 software (Premier Biosoft International, CA, United States) (Supplementary Table S3) and synthesized by China Zhongke Yutong Technology Service Company Limited. In this study, an Agilent Stratagene fluorescent quantitative PCR instrument (Mx3000P) (United States) was used to perform RT-qPCR experiments with a total amplification system of 20.0 μL. Among them, Bester SYBR Green qPCR Master Mix (Accurate, Hunan, China) 10.0 μL, PCR Forward Primer (10.0 μmol/L) 1.0 μL, PCR Reverse Primer (10.0 μmol/L) 1.0 μL, cDNA template 1.0 μL and RNase Free H_2_O 7.0 μL. Amplification procedure: 95°C for 2 min, 95°C for 3 s, 53°C for 30 s, 38 cycles, each sample was repeated three times.

### Cell differentiation

Myoblasts were cultured in 20% FBS growth medium until the cell density reached 90% and then subjected to starvation treatment for 2 h. The overexpressed plasmids and siRNAs were transfected, respectively. At the same time, a differentiation medium containing 2% HS (G-clone, Beijing, China) + 97% DMEM/F12 (BI, Israel) + 1% dual antibody (G-clone, Beijing, China) was added to induce differentiation. The transfection and differentiation times were 24, 48, and 72 h. Three biological replicates were set up at each time point. Cells were collected at the end of transfection and RNA from myoblasts was extracted to detect the expression of differentiation marker genes.

### Cell proliferation

In order to detect the effect of *PGM1* on the proliferation of myoblasts, myoblasts were seeded in a 96-well plate (Costar, United States) and 100 μL of 20% FBS medium was added to each well, about 1 × 10^4^ cells. When the degree of cell fusion reaches 70–80%, transfect the overexpression vector and siRNA. At 24, 48, and 72 h after transfection, add 10 μL of Enhanced Cell Counting Kit-8 (Beyotime, Shanghai, China) at a ratio of 1: 10 to avoid light. Cover the 96 well plate with tinfoil paper and incubate it in the cell incubator for 2 h. Measure the absorbance with a multifunctional microplate reader (BioTek, United States) at 450 nm.

The expression of cell proliferation marker gene was detected at 48 h after transfection of overexpression plasmid and siRNA and EDU was detected at 24, 48, and 72 h after transfection with BeyoClickTMEDU-594 cell proliferation detection kit (Beyotime, Shanghai, China). In short, the configured EDU working solution and medium were added to the six-well plate at 1:1 and then incubated in a cell incubator at 37°C for 2 h. After the EDU labeling cells were completed, 4% paraformaldehyde was added to the six-well plate for fixation. Add permeabilization solution to each well and incubate at room temperature for 20 min, then add 500 μL of Click reaction solution, cover the 6-well plate with tin foil paper and incubate at room temperature for 30 min in the dark. Finally, add 1 × Hoechst33342 solution and incubate in the dark for 10 min, then the fluorescence detection can be performed. Observe and take pictures with an inverted fluorescence microscope, the red fluorescence is the proliferating cells and the blue fluorescence is the nucleus.

### Cell apoptosis

The expression of apoptosis marker gene of myoblasts was detected by transfection of *PGM1* overexpression vector and interfering small RNA at 48 h. At the same time, apoptosis was detected by Annexin V-mCherry/SYTOX Green apoptosis detection kit (Beyotime, Shanghai, China) at 24, 48 and 72 h. According to the manufacturer’s instructions, myoblasts were digested and collected with trypsin (Hyclone, United States). After centrifugation, 194 μL Annexin V-mCherry Binding Buffer resuspension cells were added and then 5 μL Annexin V-mCherry and 1 μL SYTOX Green were added and incubated at room temperature for 20 min. After incubation, the cells were collected by centrifugation and resuscitated with 50 μL Annexin V-mCherry Binding Buffer. After smear, the cells were observed under fluorescence microscope.

### ATP content detection

Myoblasts were collected and centrifuged to obtain the cell pellet according to the requirements of the ATP content assay kit (Nanjing Jiangcheng Bioengineering Institute) and 500 μL of hot double-distilled water was added and then the cell suspension was heated in a boiling water bath for 10 min, then removed, mixed and extracted for 1 min, which could be used to determine the intracellular ATP levels in different treatment groups. The absorbance of each group was detected by UV spectrophotometer (Shanghai Yuanxi) at 636 nm and the ATP content was calculated according to the formula: ATP concentration (μmol/uHb) = OD value of measurement - OD value of the control group/standard OD value - blank OD value * concentration of the standard substance (1*10^3^ μmol/L) * dilution of the sample before the measurement/protein concentration of the test sample (gprot/L).

### Effect of *PGM1* on metabolites in the purine metabolic pathway

Overexpression plasmids and siRNA were transfected when the confluence of myoblasts reached 70% and were detected according to the methods of IMP Detection Kit (Nanjing Jiangcheng Bioengineering Institute) and HX, UA Enzyme Linked Immunoassay Kit (Jiangsu Enzyme). Cells were digested with trypsin, collected and centrifuged and the cell pellet was resuspended in PBS (Hyclone, United States) and centrifuged at 1,000 rpm for 10 min, repeated twice. Add 500 μL of PBS to resuspend the cells and manually homogenize the cells in ice-water mixture for 3 min, then add the samples and standards into the enzyme labeling plate according to the kit instructions and put it into an electrically heated thermostatic incubator (Langan, Shanghai, China) at 37°C for 30 min; wash the plate for 5 times, add the enzyme labeling reagent and incubate for 30 min at 37°C, wash the plate for 5 times and then add the color development solution A and B in the dark and incubate for 30 min at 37°C and finally add the termination solution. Finally, add the termination solution. The absorbance values of each group were detected at 450 nm with a multifunctional microplate reader (BioTek, United States) and a standard regression curve was plotted to calculate the metabolite content.

### *PGM1* in the synthesis of IMP in the purine metabolic pathway

When the myoblasts confluence reached 70%, 2 μL of 6-mercaptopurine (MCE, Shanghai, China), an inhibitor of purine metabolic pathway, was added dropwise and an equal amount of 100% DMSO was added as a control. The inhibitor was added every 24 h. Then *PGM1* overexpression vector or siRNA was added with 6-mercaptopurine and 100% DMSO was used as a negative control in both groups and RNA was extracted after 48 h to detect the synthesis of IMP by *PGM1* in the purine metabolic pathway.

### Data analysis

The method of 2^-ΔΔCt^ was used to calculate the relative expression level of genes and GraphPad Prism 8 (GraphPad Software Inc., San Diego, California, United States) was used to perform one-way ANOVA on the significance of differences. *p* < 0.05 indicates significant differences, *p* < 0.01 indicates extremely significant differences.

## Results

### Combined proteomics and metabolomics analysis

**Figure 1:**
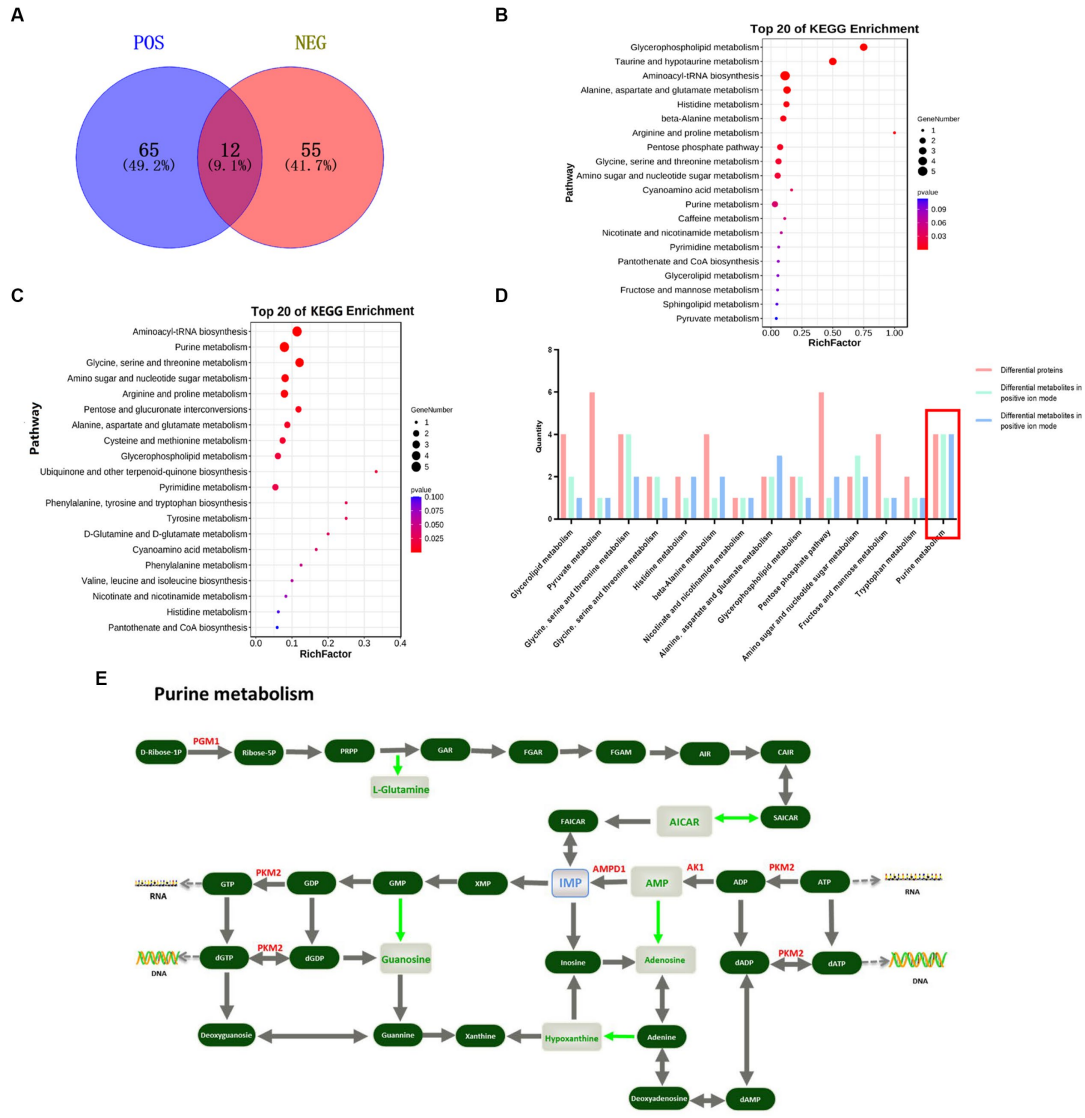
Combined protein and metabolome analyses screening for key functional proteins associated with IMP specific deposition. **(A)** Venn diagram of differential metabolites obtained by screening in positive and negative ion mode, blue is the differential metabolite in positive ion mode, red is the differential metabolites; **(B)** Top 20 metabolic pathways enriched for DE metabolites in positive ion mode; **(C)** Top 20 metabolic pathways enriched for DE metabolites in negative ion mode; **(D)** Differential proteins and metabolites jointly participate in 14 metabolic pathways; **(E)** Location of differential proteins and metabolites in Purine metabolism pathway, red text represents DE proteins, green text represents DE metabolites and blue text represents IMP.

Metabolomic analysis was performed on the breast and leg muscles of Jingyuan chicken and the relative quantitative mean values of the DE metabolites for each individual are shown in Supplemental Table S4. In breast muscle, 77 DE metabolites were screened in positive ion mode, of which 36 were up-regulated and 41 down-regulated; 67 DE metabolites were screened in negative ion mode, of which 34 were up-regulated and 33 down-regulated (Supplementary Figures S1A,B). The enrichment analysis with the KEGG pathway database (23) was performed on the identified DE metabolites and the DE metabolites were mainly enriched in pathways, such as Aminoacyl-tRNA biosynthesis, Purine metabolism, Glycine, serine and threonine metabolism and Arginine and profile metabolism (Figures 1A–C). In the purine metabolism pathway, there are 6 DE metabolites downregulated, including L-Glutamine, Adenosine monophosphate (AMP), Hypoxanthine, Guanosine, AICAR and Adenylsuccinic acid (Figure 1E).

DE proteins and DE metabolites were mapped using the KEGG pathway as a connecting hub. DE proteins and DE metabolites involved 14 metabolic pathways, of which the more classical ones, such as lipid metabolism, amino acid biosynthesis, purine metabolism and glucose metabolism, were closely related to muscle growth and meat flavor. A total of 14 metabolic pathways were involved, most of which were related to meat quality traits, among which purine metabolic pathway accounted for a larger proportion of DE proteins and DE metabolites (Figure 1D). In the purine metabolic pathway, DE protein *PGM1* functioned at the initial site of IMP *de novo* synthesis, with an up-regulation of the adjacent L-glutamine. *PKM2* was down-regulated in the IMP salvage pathway and co-regulated the DE metabolites adenosine monophosphate (AMP), hypoxanthine and guanosine with *AK1*. In addition, downregulation of *AMPD1* may affect the DE metabolites AICAR and adenosuccinate (Figure 1E). Notably, in contrast to other DE proteins, *PGM1* catalyzes the generation of ribulose-5P from D-ribulose-1P in the first step of the IMP *de novo* synthesis pathway, thereby initiating IMP synthesis and metabolism.

### *PGM1* promotes myoblast differentiation

**Figure 2:**
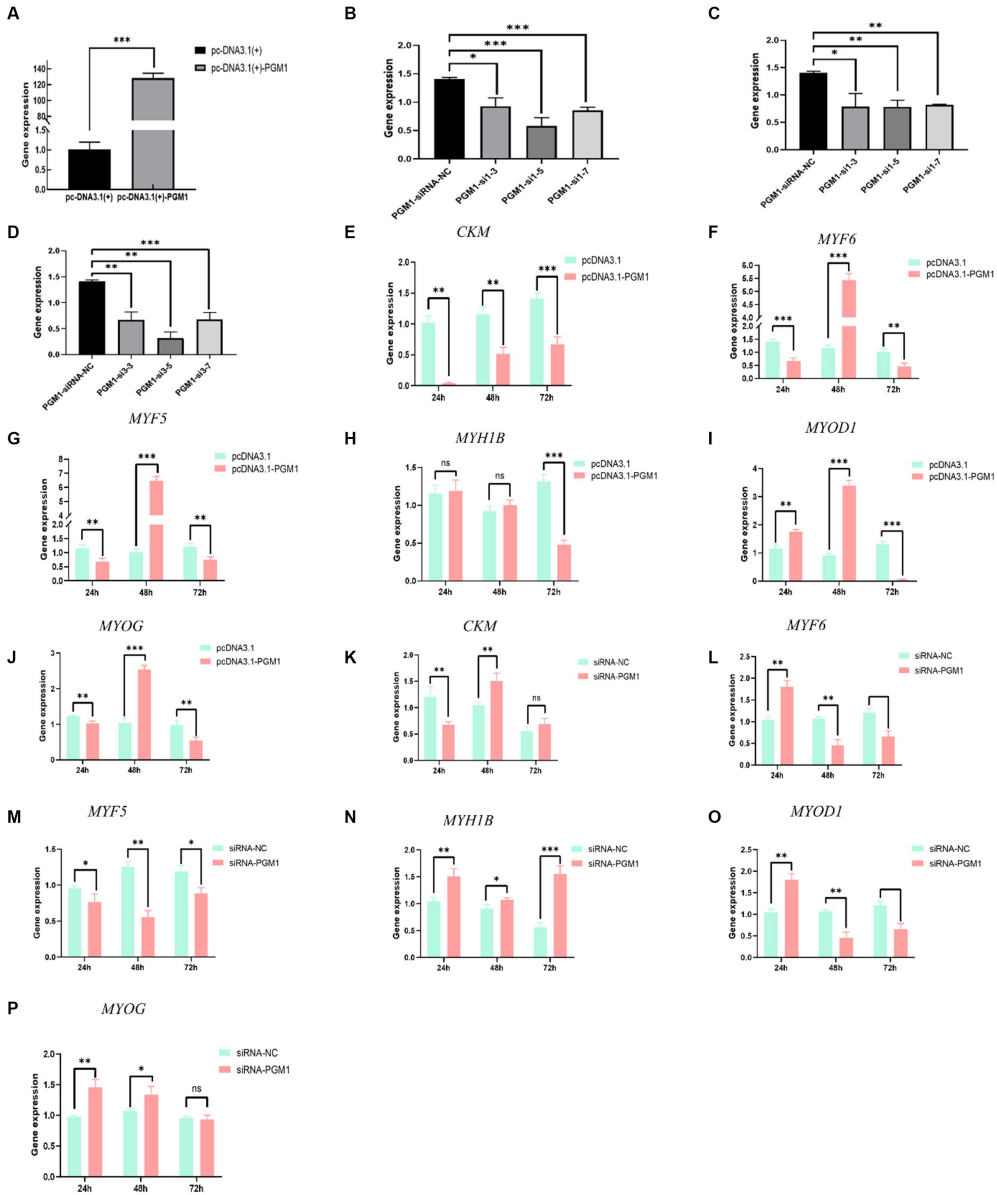
Effect of *PGM1* overexpression and interference on differentiation of myoblasts. **(A)** The overexpression efficiency of *PGM1* in myoblasts; **(B–D)** The interference efficiency of three interfering *PGM1* interfering small RNAs in myoblasts; **(E–L)** Effect of overexpression of *PGM1* on the expression of *CKM*, *MYF6*, *MYF5*, *MYH1B*, *MYOD1*, and *MYOG*; **(I–P)** Effect of interference with *PGM1* on the expression of *CKM*, *MYF6*, *MYF5*, *MYH1B*, *MYOD1*, and *MYOG.*

To verify the transfection efficiency of the *PGM1* overexpression vector and interfering small RNAs, the overexpression plasmid and three siRNAs were transfected into myoblasts at different concentration gradients. Overexpression was increased 120-fold in the pcDNA3.1-*PGM1* group compared to the pcDNA3.1 group (Figure 2A). *PGM1* showed the highest interference efficiency of more than 90% for the third siRNA compared to the negative control (NC). *PGM1*-siRNA3 was selected as the best interfering fragment (Figures 2B–D). Then expression of differentiation marker genes was assayed during induction of myoblast differentiation. Compared with the control group, the expression levels of the differentiation marker genes muscle-specific creatine kinase (*CKM*), myogenic regulatory factor 6 (*MYF6*), myogenic factor 5 (*MYF5*) and myogenin (*MYOG*) were suppressed in the overexpression group and the expression of myogenic differentiation factor 1 (*MYOD1*) was significantly elevated (*p* < 0.05), whereas the expression of fast myosin heavy chain (*MYH1B*) did not change significantly at 24 h (*p* > 0.05). And the expression of other differentiation marker genes were decreased at 48 h. The expression of differentiation marker genes showed a downward trend at 72 h (Figures 2E–J). Meanwhile, an opposite trend was observed in the interference group compared with the NC-siRNA group (Figures 2K–P). The differentiation and development of myoblasts into multinucleated myotubes is controlled at multiple levels by a number of key transcription and regulatory factors (24). The *CKM* and myogenesis regulatory factors (*MYFs*) family of genes play important regulatory roles in initiating and maintaining the process of skeletal myoblast differentiation and are considered to be hallmark factors that promote myoblast differentiation (25, 26). Based on these results we conclude that *PGM1* promotes myoblast differentiation.

### *PGM1* promotes the proliferation of myoblasts

**Figure 3:**
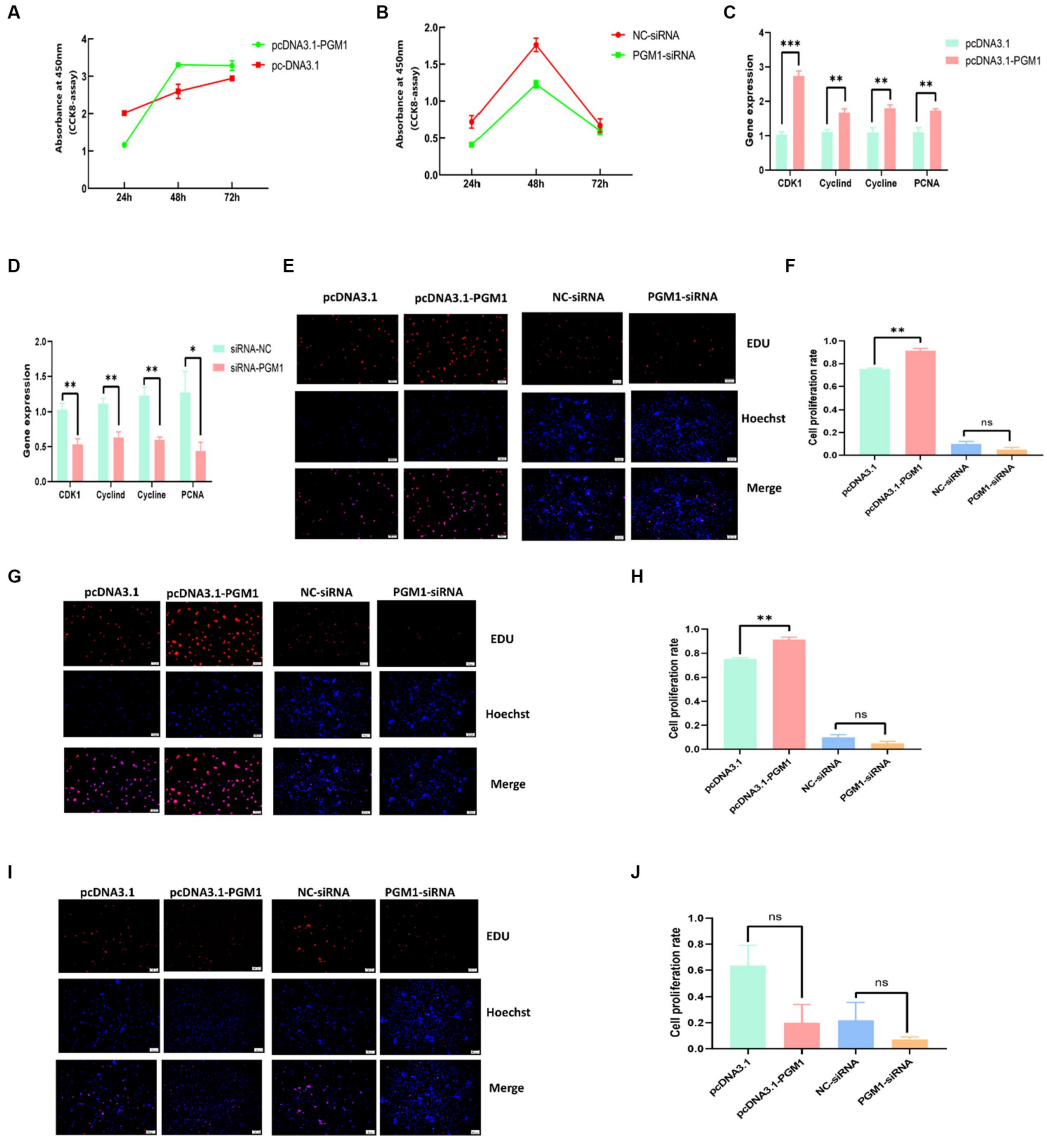
Effect of *PGM1* on proliferation of myoblasts. **(A,B)** Detection of proliferation viability of myoblasts by overexpression and interference with *PGM1*; **(C,D)** Expression levels of proliferation marker genes by overexpression and interference with *PGM1*; **(E)** Cell proliferation at 24 h after overexpression and interference with *PGM1* by EDU; **(F)** Myoblasts proliferation rate at 24 h after overexpression and interference with *PGM1*; **(G)** Cell proliferation at 48 h after overexpression and interference with *PGM1* by EDU; **(H)** Myoblasts proliferation rate at 48 h after overexpression and interference with *PGM1*; **(I)** Cell proliferation at 72 h after overexpression and interference with *PGM1* by EDU; **(J)** Myoblasts proliferation rate at 72 h after overexpression and interference with *PGM1.*

We examined the proliferative activity of myoblasts at 24, 48, and 72 h. The proliferative activity after overexpression of *PGM1* was higher than that of the control group and the proliferative activity after interfering with *PGM1* was significantly lower than that of the NC group ((Figures 3A,B), Supplementary Table S5). Meanwhile, overexpression of *PGM1* resulted in significantly higher expression of proliferation marker genes such as *cyclin D, cyclin E, Cyclin* dependent kinases 1 (*CDK1*) and DNA polymerase cofactor (*PCNA*) (*p* < 0.01), whereas the expression of the proliferation marker genes was significantly reduced after interfering with *PGM1* (*p* < 0.05) (Figures 3C,D). At 24 h of transfection, overexpression of *PGM1* increased the proliferation rate of myoblasts, but not significantly, whereas interference with *PGM1* significantly decreased the value-added rate of myoblasts (*p* < 0.05) (Figures 3E,F). At 48 h of transfection, *PGM1* highly significantly reduced myoblasts value-added rate (*p* < 0.01), whereas down-regulation of *PGM1* cell value-added rate was reduced but not significantly (Figures 3G,H). At 72 h of transfection, *PGM1* had no significant effect on myoblasts viability compared to control (Figures 3I,J). It is well known that cell proliferation is an important life feature of organisms and cell proliferation in the form of division plays an extremely important role in the growth and development of organisms (27, 28). *Cyclin D, cyclin E, CDK1* and *PCNA* play important roles in initiating cell proliferation (29, 30). Combined with our results, we show that *PGM1* promotes the proliferation of myoblasts.

### *PGM1* inhibits myoblast apoptosis

**Figure 4:**
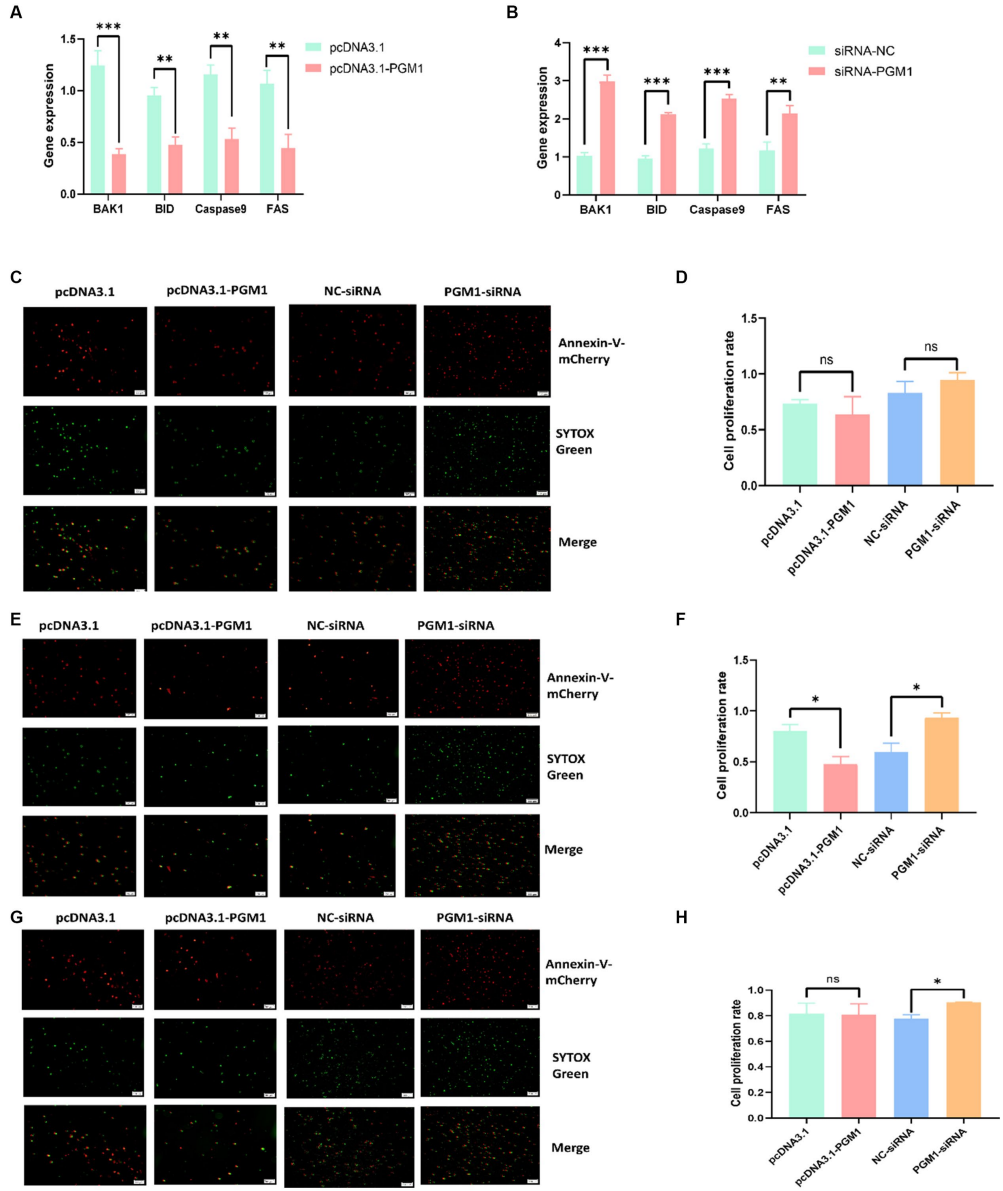
Effect of *PGM1* on the apoptosis of myoblasts. **(A,B)** Expression levels of apoptosis marker genes detected by overexpression and interference with *PGM1*; **(C)** Apoptosis detection by Annexin V-mCherry/SYTOX Green at 24 h after overexpression and interference with *PGM1*; **(D)** Apoptosis rate of myoblasts at 24 h after overexpression and interference with *PGM1*; **(E)** Apoptosis detection by Annexin V-mCherry/SYTOX Green at 48 h after overexpression and interference with *PGM1*; **(F)** Apoptosis rate of myoblasts at 48 h after overexpression and interference with *PGM1*; **(G)** Apoptosis detection by Annexin V-mCherry/SYTOX Green at 72 h after overexpression and interference with *PGM1*; **(H)** Apoptosis rate of myoblasts at 72 h after overexpression and interference with *PGM1.*

We transfected overexpression vectors and siRNA of *PGM1* into myoblasts to detect the effect of *PGM1* on apoptosis. Overexpression of *PGM1* suppressed the expression of apoptosis marker genes, whereas interference with *PGM1* greatly promoted the expression of apoptosis marker genes (Figures 4A,B). The Annexin V-mCherry/SYTOX Green apoptosis assay showed that at 24 h, the apoptosis ratio of the overexpression and interference groups did not differ from that of the control group (Figures 4C,D). After 48 h of transfection, the number of apoptotic cells in the overexpression group was significantly lower than that in the control group (*p* < 0.05) and the number of apoptotic cells in the interference group was significantly higher than that in the NC group (*p* < 0.05) (Figures 4E,F). Until 72 h, there was no significant difference in the number of apoptotic cells in the overexpression group and the number of apoptotic cells in the interference group was higher than that in the NC group (Figures 4G,H). Apoptosis has been found to be a normal physiological process indispensable for embryonic development, tissue homeostasis and organismal stability (31, 32). Antagonist-Killer Protein (*Bak1*), TNF receptor superfamily, member 6 (*FAS*), *caspase 9* and BH3-interacting domain death agonist (*BID*) genes have pro-apoptotic activity and cause apoptosis (33, 34). And our study showed that upregulation of *PGM1* suppressed the expression of apoptosis marker genes. Therefore, *PGM1* may play a negative role in the apoptotic process of myoblasts.

### *PGM1* inhibits ATP, IMP, and UA biosynthesis, increases HX biosynthesis

**Figure 5:**
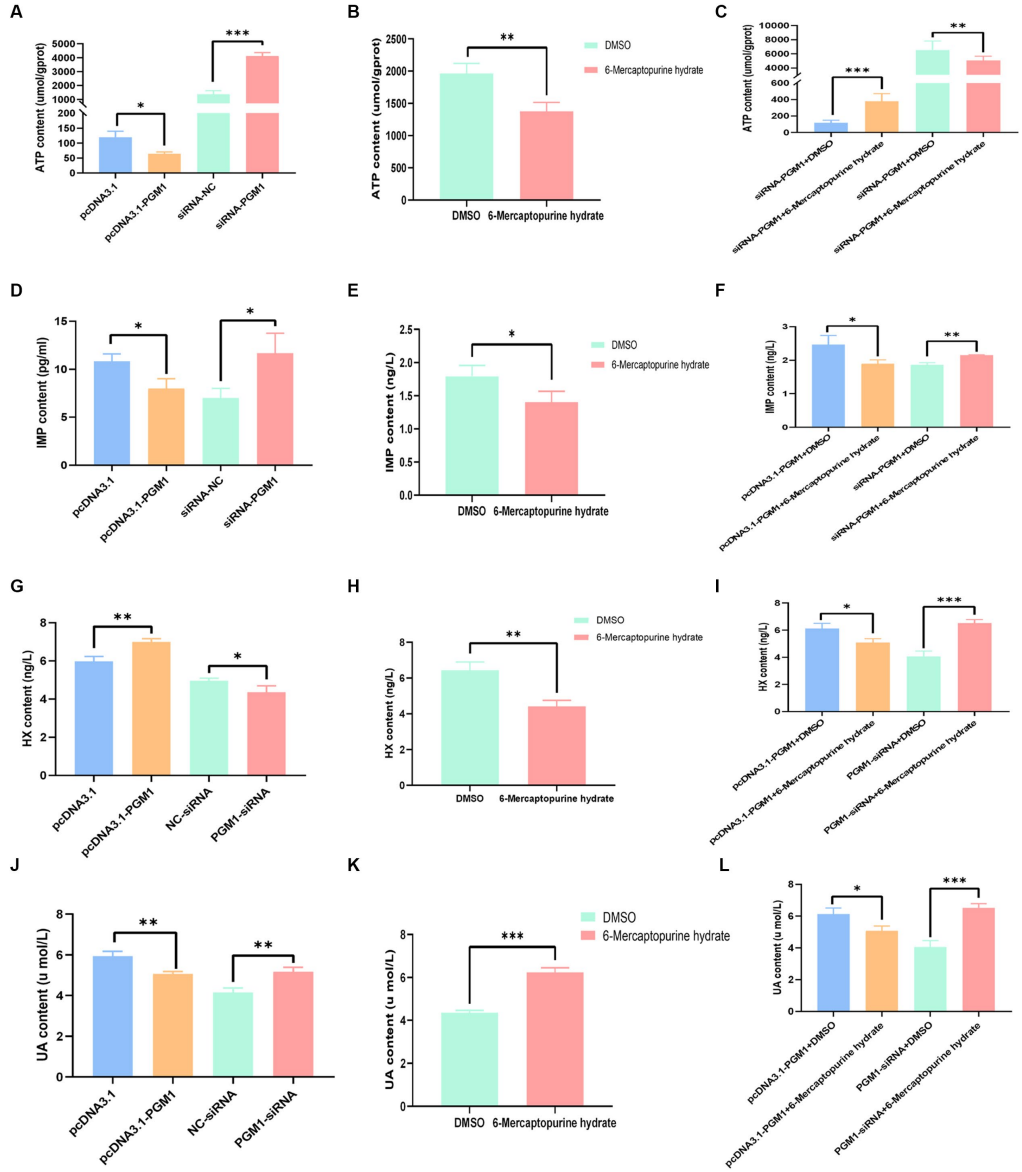
Effect of *PGM1* on ATP, IMP, HX, and UA content. **(A)** Effects of overexpression and interference with *PGM1* on ATP content; **(B)** Effect of inhibitor on ATP content; **(C)** Effects of overexpression/interference with *PGM1* and inhibitors on ATP content; **(D)** Effects of overexpression and interference with *PGM1* on IMP content; **(E)** Effect of inhibitor on IMP content; **(F)** Effects of overexpression/interference with *PGM1* and inhibitors on IMP content; **(G)** Effects of overexpression and interference with *PGM1* on HX content;**(H)** Effect of inhibitor on HX content; **(I)** Effects of overexpression/interference with *PGM1* and inhibitors on HX content; **(J)** Effects of overexpression and interference with *PGM1* on UA content; **(K)** Effect of inhibitor on UA content; **(L)** Effects of overexpression/interference with *PGM1* and inhibitors on UA content.

To investigate the effects of *PGM1* on purine metabolism, we examined the levels of the above products after overexpression and interference with *PGM1* and the results showed that overexpression of *PGM1* inhibited the biosynthesis of ATP, IMP, and UA but promoted the biosynthesis of HX compared to the control group, whereas the opposite tendency was observed for interference with *PGM1* (Figures 5A,D,G,J). The addition of inhibitors resulted in decreased ATP, IMP and HX biosynthesis and increased UA biosynthesis (Figures 5B,E,H,K). The combination of inhibitor and *PGM1* overexpression enhanced ATP biosynthesis and inhibited IMP, HX, and UA biosynthesis, whereas inhibitor and interfering *PGM1* promoted IMP, HX, and UA and inhibited ATP (Figures 5C,F,I,L).

### Effects of *PGM1* in the purine metabolism pathway

**Figure 6:**
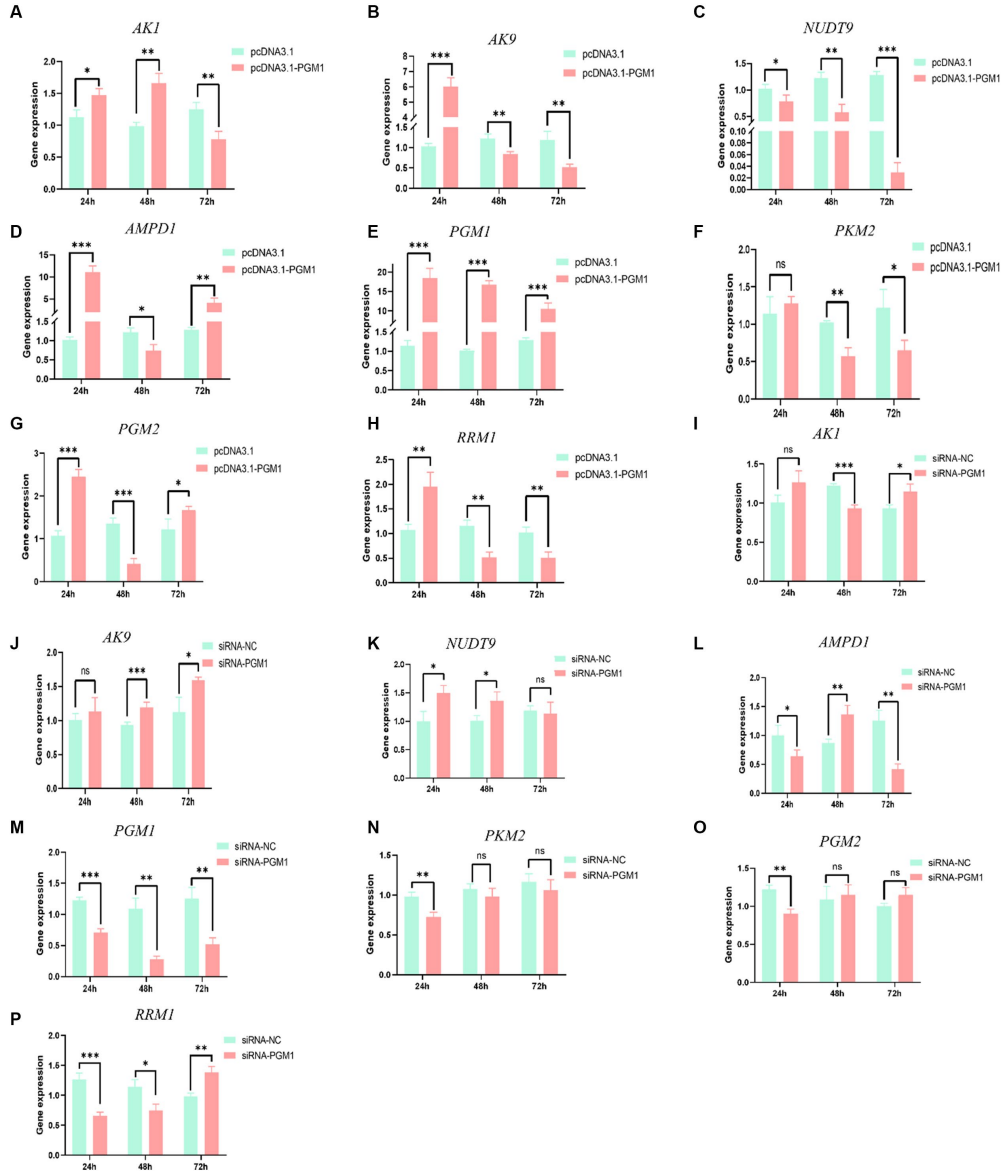
The effect of overexpression and interference with *PGM1* in the purine metabolism pathway. **(A–H)** The effect of overexpression of *PGM1* on the expression levels of *AK1*, *AK9*, *AMPD1*, *NUDT9*, *PGM1*, *PKM2*, *PGM2*, and *RRM1*; **(I–P)** The effect of interference with *PGM1* on the expression levels of *AK1*, *AK9*, *AMPD1*, *NUDT9*, *PGM1*, *PKM2*, *PGM2*, and *RRM1.*

Analysis of the upstream and downstream of the *PGM1* gene in the purine metabolic pathway showed that after overexpression of *PGM1* for 24 h, the expression of *AK1*, adenylate kinase 9 (*AK9*), nucleotide hydrolase 9 (*NUDT9*), *PGM1*, phosphoglucose aminotransferase 2 (*PGM2*) and ribonucleotide reductase M1 (*RRM1*) was significantly increased (*p* < 0.05) and that *AMPD1* expression was significantly decreased (*p* < 0.05), while the expression of *PKM2* did not change (*p* > 0.05). At 48 h, *AK1* and *PGM1* still showed an increasing trend, while *AK9*, *AMPD1*, *NUDT9*, *PKM2*, *PGM2*, and *RRM1* showed a significant decreasing trend. At 72 h, the expression levels of *AK1*, *AK9*, *AMPD1*, *PKM2*, and *RRM1* decreased, whereas those of *NUDT9*, *PGM1*, and *PGM2* increased (Figures 6A–H). Interference with *PGM1* showed an opposite trend, suggesting that *PGM1* expression enhances the expression of both upstream and downstream genes in the purine metabolic pathway (Figures 6I–P).

### *PGM1* enhances IMP synthesis through purine metabolism pathway

**Figure 7:**
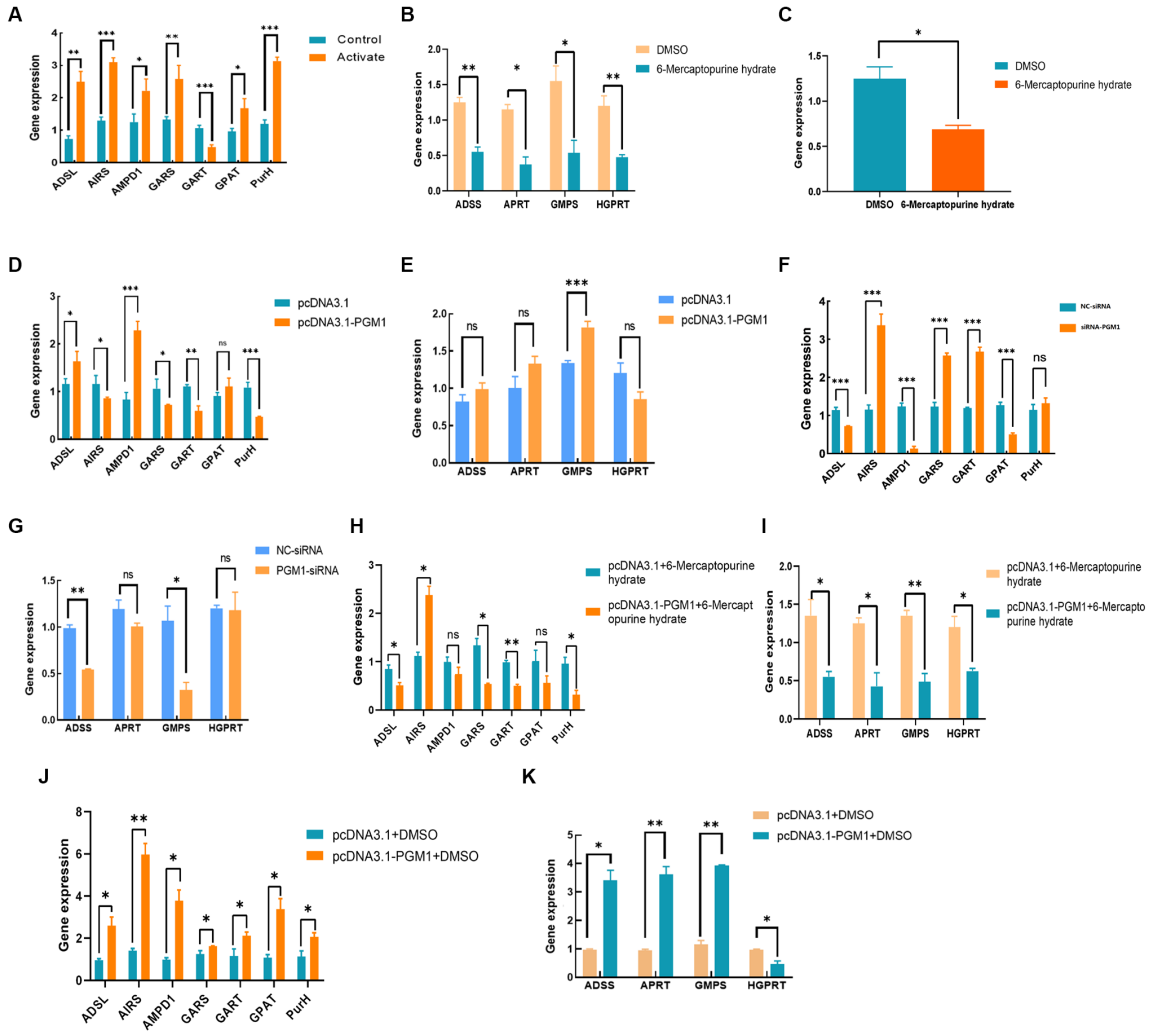
Effect of *PGM1* on IMP synthesis through purine metabolic pathway. **(A,B)** Effect of adding inhibitor on the expression level of IMP synthesis pathway; **(C)** Effect of inhibitor on *PGM1* expression; **(D,E)** Effect of overexpressing *PGM1* on IMP synthesis pathway; **(F,G)** Effect of interfering with *PGM1* on IMP synthesis pathway; **(H–K)** Effect of *PGM1* and inhibitor together on IMP synthesis pathway.

The addition of inhibitors promoted the expression of key genes in the IMP *de novo* synthesis pathway and inhibited the expression of key genes in the rescue synthesis pathway and reduced the expression of *PGM1* gene (Figures 7A–C). The control group, overexpressing *PGM1* led to a significant increase in the expression of key genes of the *de novo* synthesis synthesis pathway *ADSL*, *AMPD1*, and *GPAT*, while the expression of *AIRS*, *GARS*, and *PurH* showed a downward trend. The expression level of *HGPRT*, a key gene in the remedial synthesis pathway, was lower than that of the control group, while the expression levels of *ADSS*, *APRT*, and *GMPS* were increased (Figures 7D–G). Interference with *PGM1* has the opposite effect. In addition, under the combined effect of overexpression of *PGM1* and inhibitor, the expression levels of key genes of *de novo* synthesis synthesis and salvage synthesis of IMP showed a downward trend, while the opposite was true for the overexpression of *PGM1* and DMAO groups (Figures 7H–K). This indicates that *PGM1* promotes the expression of key genes of IMP *de novo* synthesis synthesis pathway and remedial synthesis pathway and then regulates the specific deposition of IMP.

## Discussion

IMP is an indicator for evaluating the flavor of livestock and poultry meat, which is widely present in the muscle tissues of livestock and poultry. In-depth study of the synthesis and metabolism mechanism of IMP can help to improve the quality of livestock and poultry meat. A study analyzed and identified key genes affecting the formation of meat flavor in four developmental stages of Beijing-You chicken, among which 310 significantly changed metabolites (SCMs) and 7,225 DE genes (DEGs) were identified and KEGG enrichment analyses showed that SCMs and DEGs were enriched in amino acid, lipid and IMP metabolic pathways (35). Analyses of meat quality and breast muscle of Lueyang Black-Bone Chickens from different rearing methods revealed that pH, shear, IMP, palmitic acid and linoleic acid values were significantly higher (*p* < 0.05) in the free range group than in the cage rearing group. Meanwhile, 291, 131, and 387 DE genes were associated with muscle development and amino acid metabolic pathways in the three comparative groups (caged vs. free range, flat-netted vs. caged and flat-netted vs. free range), respectively (36). In an earlier study, we used RNA-seq to analyze the gene expression of Jingyuan chickens of different rearing methods and different genders. The results showed that the breast muscle IMP content of indigenous chickens was higher than that of caged chickens and the breast muscle IMP content of hens was higher than that of roosters. The expression of glutamate aminoligase was positively but not significantly correlated with the IMP content of caged and free-ranging chickens and the expression of phosphodiesterase 10A was also positively correlated with the IMP content of caged and free-ranging chickens (37). In addition, the DE gene *AK1* was screened by transcriptomics and correlated with the IMP content of Jingyuan chickens. Its content was negatively correlated with the IMP content, while its expression in the leg muscles of roosters was positively correlated with the IMP content. Based on the above research basis, this study combined proteomics and metabolomics to screen four DE proteins, *AK1*, *AMPD1*, *PKM2*, and *PGM1* and six DE metabolites, hypoxanthine, guanosine, L-glutamine, AICAR, AMP and adenylate, which were down-regulated in the purine metabolic pathway. The results suggest that the interactions of four DE proteins and six different metabolites have important effects in the synthesis and catabolic pathways of IMP. Therefore, we hypothesized that the differences in IMP content in muscle tissues of Jingyuan chickens were due to the regulation of different proteins and different metabolites.

As a key enzyme in glycolysis, gluconeogenesis and catabolism, *PGM1* is a key regulator of carbohydrate metabolism in mammals (37). An increasing number of studies have shown that *PGM1* plays an important role in physiological processes such as cell proliferation and programmed death (18). It has been reported that the infinite proliferation and apoptosis of many tumor cells and cancer cells in the human body are closely related to the *PGM1* gene and that *PGM1* provides cancer cells with energetic substances, such as glycogen, to support growth, metastasis and invasion of cancer cells (38, 39). The effect of the expression level of *PGM1* on cancer cells varies depending on the cell type and knockdown or deletion of the *PGM1* gene inhibits glycogen synthesis and disruption of glycolysis, which promotes the proliferation and growth of hepatocellular carcinoma cells (40–42). In addition, *PGM1* expression in cancer cells inhibits cancer cell proliferation (43). Notably, it has been noted that high expression of *PGM1* induces the survival and proliferation of lung cancer cells, whereas reduced expression of *PGM1* decreases the proliferation of lung cancer cells (44). However, there are not many reports on the regulation of chicken muscle development by the *PGM1* gene. Our results showed that up-regulation of *PGM1* promoted the differentiation and proliferation of myoblasts and inhibited the apoptotic process of myoblasts, which is consistent with the findings of previous studies. This suggests that *PGM1* plays an important role in promoting the growth and development of Jingyuan chicken muscle.

**Figure 8:**
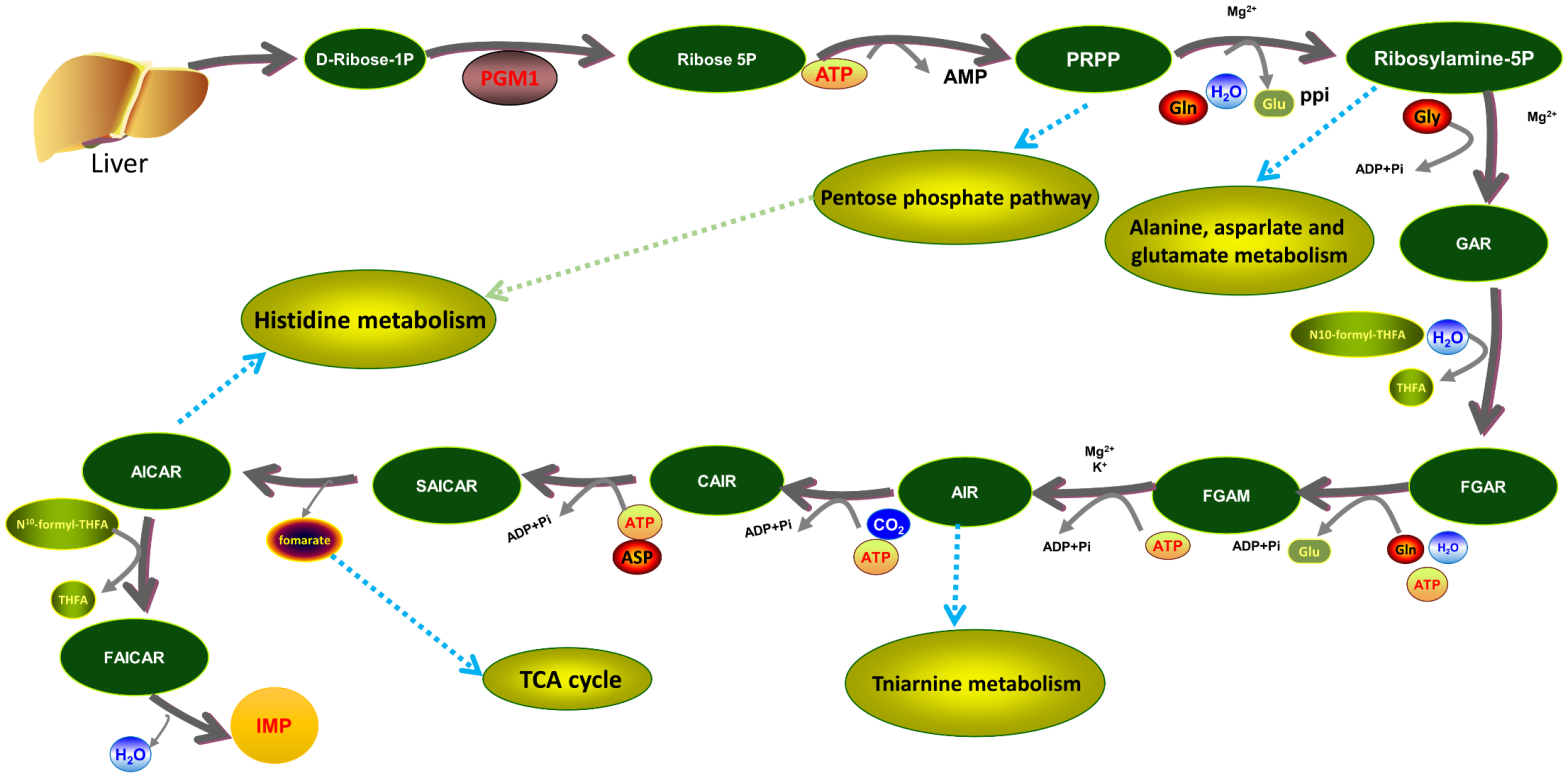
The position of *PGM1* in the *de novo* and synthesis pathways of IMP.

In the purine metabolism pathway, *PGM1* plays a crucial role in the initiation of the IMP *de novo* synthesis pathway. It catalyzes the production of ribose-5P from D-ribose-1P in the first step of the IMP *de novo* pathway, thereby initiating IMP synthesis and metabolism (Figure 8). It was found that the *PGM1* gene is in the same chromosomal region as many key genes affecting meat flavor and pork growth and molecular markers that may significantly affect pig leanness may be present on Exon3 of *PGM1*, suggesting that the *PGM1* gene is closely related to the production and development of animal muscle (21). Analysis of polymorphisms and mRNA levels of the *PGM1* gene in highland Tibetan and Yorkshire pigs revealed that the expression of the *PGM1* gene in the dorsal adipose tissue of Tibetan pigs was lower than that of Yorkshire pigs and it was hypothesized that the *PGM1* gene might be involved in the regulation of muscle growth and fat deposition in pigs (45). However, there are few reports on the mechanisms by which *PGM1* regulates chicken meat quality and flavor. One study identified and characterized fat deposition genes in chickens and found that carbohydrate metabolism genes (*MGAT4B*, *XYLB*, *GBE1*, *PGM1*, and *HKDC1*) were higher in fast-growing white-backed stonechucks than in slow-growing Xinghua chickens and that the parallel expression patterns of these functionally related genes provided strong evidence (46). In this study, functional validation of the *PGM1* gene showed that overexpression of *PGM1* repressed genes upstream and downstream of the purine metabolism pathway, whereas it enhanced the expression of genes key to the IMP *de novo* and salvage synthesis pathways. Detection of marker metabolites in the purine metabolic pathway revealed that high expression of *PGM1* inhibited the biosynthesis of ATP, IMP, and UA but promoted the biosynthesis of HX, these findings suggest that *PGM1* regulates IMP synthesis and metabolism through the purine metabolic pathway.

## Conclusion

In summary, our results indicate that *PGM1* expression can promote the proliferation and differentiation of myoblasts as well as the *de novo* and salvage synthesis pathways of IMP, which in turn affects the meat flavour of Jingyuan chicken.

## Data availability statement

The original contributions presented in the study are included in the article/Supplementary material, further inquiries can be directed to the corresponding author.

## Ethics statement

The animal studies were approved by the Animal Welfare Committee of Ningxia University and was conducted according to the Guidelines of Animal Use of the Committee of the Ministry of Agriculture of China (Beijing, China). The studies were conducted in accordance with the local legislation and institutional requirements. Written informed consent was obtained from the owners for the participation of their animals in this study.

## Author contributions

WZ: Data curation, Methodology, Software, Validation, Writing – original draft, Writing – review & editing. ZC: Methodology, Validation, Visualization, Writing – original draft, Writing – review & editing. CW: Software, Visualization, Writing – original draft. XM: Software, Writing – original draft. BY: Conceptualization, Data curation, Formal Analysis, Writing – review & editing. XF: Conceptualization, Data curation, Formal Analysis, Writing – original draft. TZ: Investigation, Resources, Supervision, Writing – review & editing. YG: Supervision, Writing – review & editing. JZ: Funding acquisition, Project administration, Resources, Supervision, Writing – review & editing.
