# Supplementary material for: Functional identification of PGM1 in the regulating development and depositing of inosine monophosphate specific for myoblasts
Source: Front Vet Sci. 2023 Dec 18;10:1276582. doi: 10.3389/fvets.2023.1276582 (PMC10758172; doi:10.3389/fvets.2023.1276582)
Supplement: Supplementary file 1 [file Data_Sheet_1.zip › Figure S1.DOCX]

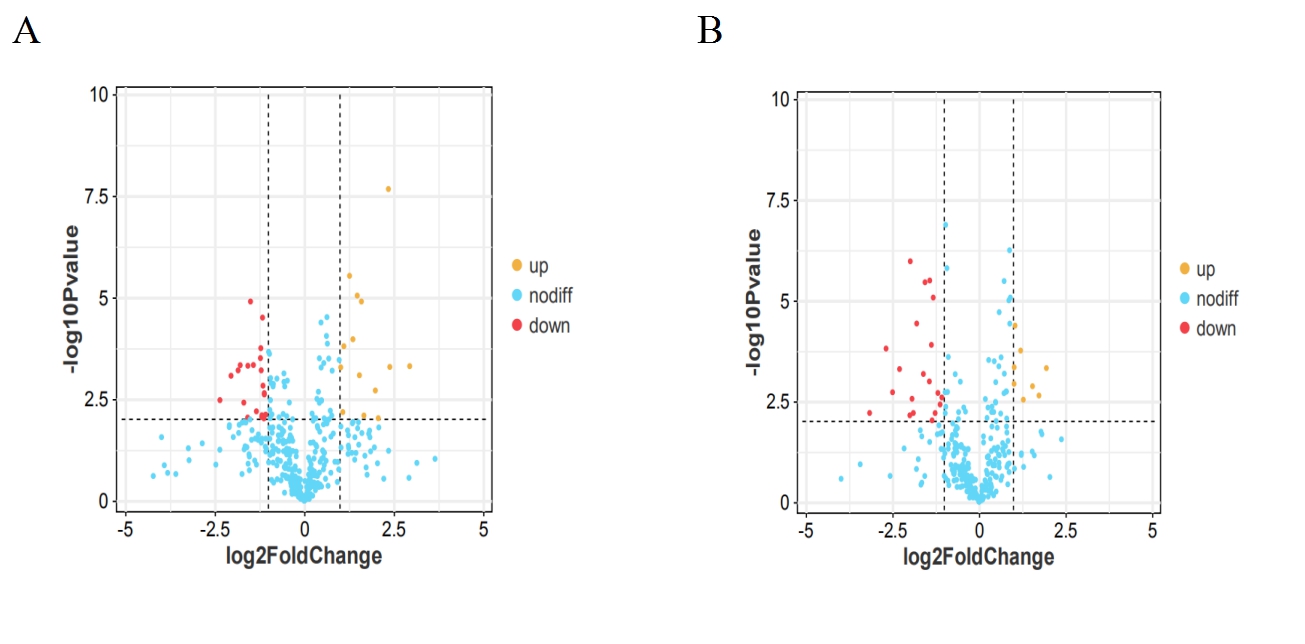


Supplementary Material Figure 1

**(A)** Differential metabolites screened in positive ion mode; **(B)** Differential metabolites screened in negative ion mode, each point in the volcano plot represents a differential metabolite, red is the down-regulated differential metabolite, yellow is the up-regulated differential metabolite;
