# Supplementary material for: Functional identification of PGM1 in the regulating development and depositing of inosine monophosphate specific for myoblasts
Source: Front Vet Sci. 2023 Dec 18;10:1276582. doi: 10.3389/fvets.2023.1276582 (PMC10758172; doi:10.3389/fvets.2023.1276582)
Supplement: Supplementary file 1 [file Data_Sheet_1.zip › Table S3.DOCX]

Supplementary Material

# Table3. Primers for related genes

| Gene | Gene entry number | Primer sequence (5′→3′) | Product length/bp |
| --- | --- | --- | --- |
| *β-actin* | L08165.1 | F: 5' ATGGACTCTGGTGATGGTGTTAC 3' | 185 |
|  |  | R: 5' TCGGCTGTGGTGGTGAAG 3' |  |
| *PKM2* | NM_205469.2 | F: 5' GCAGGGACCGCTTTCAT 3' | 107 |
|  |  | R:5' CTGGCAATGGTTGGCTC 3' |  |
| *PGM1* | NM_001038693.3 | F: 5' ATCACTGGCAGAAGTATGG 3' | 170 |
|  |  | R: 5' GGCTTTCTCAACCGTGTA 3' |  |
| *RRM1* | NM_001030837.2 | F: 5' GCCCAAATGAGTGTCCT 3' | 132 |
|  |  | R: 5' ATTCGATGATGGCGTAC 3' |  |
| *AK9* | XM_040668586.1 | F: 5' CAGTCAATGGGTGTTCG 3' | 135 |
|  |  | R: 5' TCGGTATCTGGGTGCTA 3' |  |
| *PGM2* | NM_001031383.2 | F: 5' CTGAAGAAGGCAAAGGT 3' | 184 |
|  |  | R: 5' GTGAAGATCCACCAACC 3' |  |
| *NUDT9* | XM_040669698.1 | F: 5' GCTGTAACGCCAAGAAT 3' | 168 |
|  |  | R: 5' ACTGAGGTCCAGACAAAA 3' |  |
| *AMPD1* | XM_003642728 | F: 5' TACCCAGGATTTATGATGT 3' | 108 |
|  |  | R:5' CTTGAGGATTGACAGTTG 3' |  |
| *AK1* | NM_205109.3 | F:5' CTCCAAGGGTTTCCTCATT 3' | 185 |
|  |  | R:5' TCTTGATGGTCTCCTCGTT 3' |  |
| *MYOD1* | NM_204214.3 | F:5' CTGCCCAAGGTGGAGAT 3' | 274 |
|  |  | R:5' CGAGGCTGGAAACAACA 3' |  |
| *MYOG* | NM_204184.2 | F:5' ATGACCAGGCAGAGGACC 3' | 166 |
|  |  | R:5' TCCAGCATCACCATCCC 3' |  |
| *MYH1B* | NM_204228.4 | F:5'GGGCTTTACTGCCGATGA 3' | 110 |
|  |  | R:5'CTGGCTCTGCTTGCTCTTC 3' |  |
| *MRF6* | NM_001030746.3 | F：5' TCCGACTTCCTGAGCACCT 3' | 197 |
|  |  | R: 5' TTCTCCACCGCCTCTTCC 3' |  |
| *MYF5* | NM_001030363.2 | F: 5' GGAGGAGGCTGAAGAAAGTGAA 3' | 174 |
|  |  | R: 5' GTCCCGGCAGGTGATAGTAGTT 3' |  |
| *CKM* | NM_205507.1 | F: 5' ACCTGGGTTACATCCTGACG 3' | 296 |
|  |  | R: 5' GTCTATGGGCTGGTTCTGCT 3' |  |
| *Cycline* | BI390175 | F: 5'GGTTGTTGGCATCAGTAAAG 3' | 214 |
|  |  | R:5'TATTACCTCATCTTCCTCGTTG 3' |  |
| *Cyclind-1* | NM_205381.2 | F: 5'CAGAAGTGCGAAGAGGAAGT 3' | 189 |
|  |  | R:5'GATGGAGTTGTCGGTGTAAAT 3' |  |
| *PCNA* | NM_204170.3 | F: 5'GACTCCTCGCACGTCTCCCT 3' | 161 |
|  |  | R:5'TCCGCATTGTCTTCTGCTCTG 3' |  |
| *CDK1* | NM_205314.2 | F: 5'TGCAAGGCTGTACCTCATT 3' | 174 |
|  |  | R:5'ACTCTTAACACGCGAACGA 3' |  |
| *BAK1* | NM_001030920.3 | F: 5'GTTCGACAGCGGCATTA 3' | 158 |
|  |  | R:5'TGCCTTGCTGGTAGACG 3' |  |
| *FAS* | NM_001199487.2 | F: 5'GTTCGTCATCACCGTCTATCG 3' | 215 |
|  |  | R:5'TTCGTAGGCTCCTCCCATC 3' |  |
| *BID* | NM_204552.3 | F: 5'TCACAGGCAGTGGAAGGA 3' | 196 |
|  |  | R:5'TGTGGAAGTGTTGGCTGAT 3' |  |
| *Caspase9* | BG711928 | F: 5'CGAAGGAGCAAGCACGA 3' | 187 |
|  |  | R:5'AGGTTGGACTGGGATGGAC 3' |  |
| *AIRS* | FJ977573.1 | F: 5' GACCTGGTTGCTATGTG 3' | 250 |
|  |  | R: 5' TTTGCCCTCGCTCTACT 3' |  |
| *GART* | NM_001001469.2 | F: 5' ATTGGTTATCGGGCTAT 3' | 180 |
|  |  | R: 5' TTTCAAATCAAAGAGGC 3' |  |
| *GPAT* | EU049888.1 | F: 5' GAAGGGCTTGTATCGTC 3' | 174 |
|  |  | R: 5' ATGAACCAATGTCCACC 3' |  |
| *GARS* | NM_001396375.1 | F: 5' AAATCGTCCCAAATGTC 3' | 217 |
|  |  | R: 5' TTCCTGGTGAGTGCTTC 3' |  |
| *ADSL* | NM_205529.2 | F: 5' GGACTGCCATGAGAAGAT 3' | 140 |
|  |  | R: 5' AAAGGCTGTCAAGGTGTT 3' |  |
| *PurH* | AY787803.1 | F: 5' CCCTGACAGTGGTGAAT 3' | 288 |
|  |  | R: 5' CAGACAAGGCAATGAAGT 3' |  |
| *HGPRT* | NM_204848.2 | F: 5' ACTATGACTCTACCGACTAT 3' | 277 |
|  |  | R: 5' TAACTGGCTGCTTCTTG 3' |  |
| *APRT* | NM_001277647.4 | F: 5' CCCGTGGCTTCCTCATA 3' | 150 |
|  |  | R: 5' CATCGCTCTGGATTTCG 3' |  |
| *ADSS* | NM_001031521.2 | F: 5' CTAATGGGATGCGTTCA 3' | 167 |
|  |  | R：5' CAGGGACATAAGGGCTA 3' |  |
| *GMPS* | NM_001006556.1 | F: 5' AAAGCCACCAGGAACTA 3' | 296 |
|  |  | R: 5' ATGGGAAGAATCAGTAAGA 3' |  |
